# Delayed global feedback in the genesis and stability of spatiotemporal excitation patterns in paced biological excitable media

**DOI:** 10.1371/journal.pcbi.1007931

**Published:** 2020-10-05

**Authors:** Zhen Song, Zhilin Qu

**Affiliations:** 1 Department of Medicine, David Geffen School of Medicine, University of California Los Angeles, Los Angeles, California, United States of America; 2 Peng Cheng Laboratory, Shenzhen, China; 3 Department of Computational Medicine, David Geffen School of Medicine, University of California Los Angeles, Los Angeles, California, United States of America; University of California San Diego, UNITED STATES

## Abstract

Biological excitable media, such as cardiac or neural cells and tissue, exhibit memory in which a change in the present excitation may affect the behaviors in the next excitation. For example, a change in calcium (Ca^2+^) concentration in a cell in the present excitation may affect the Ca^2+^ dynamics in the next excitation via bi-directional coupling between voltage and Ca^2+^, forming a delayed feedback loop. Since the Ca^2+^ dynamics inside the excitable cells are spatiotemporal while the membrane voltage is a global signal, the feedback loop is then a delayed global feedback (DGF) loop. In this study, we investigate the roles of DGF in the genesis and stability of spatiotemporal excitation patterns in periodically-paced excitable media using mathematical models with different levels of complexity: a model composed of coupled FitzHugh-Nagumo units, a 3-dimensional physiologically-detailed ventricular myocyte model, and a coupled map lattice model. We investigate the dynamics of excitation patterns that are temporal period-2 (P2) and spatially concordant or discordant, such as subcellular concordant or discordant Ca^2+^alternans in cardiac myocytes or spatially concordant or discordant Ca^2+^ and repolarization alternans in cardiac tissue. Our modeling approach allows both computer simulations and rigorous analytical treatments, which lead to the following results and conclusions. When DGF is absent, concordant and discordant P2 patterns occur depending on initial conditions with the discordant P2 patterns being spatially random. When the DGF is negative, only concordant P2 patterns exist. When the DGF is positive, both concordant and discordant P2 patterns can occur. The discordant P2 patterns are still spatially random, but they satisfy that the global signal exhibits a temporal period-1 behavior. The theoretical analyses of the coupled map lattice model reveal the underlying instabilities and bifurcations for the genesis, selection, and stability of spatiotemporal excitation patterns.

## Author summary

Understanding the mechanisms of pattern formation in biological systems is of great importance. Here we investigate the dynamical mechanisms by which delayed global feedback affects excitation pattern formation and stability in periodically-paced biological excitable media, such as cardiac or neural cells and tissue. We focus on the formation and stability of the temporal period-2 and spatially in-phase and out-of-phase excitation patterns. Using models of different levels of complexity, we show that when the delayed global feedback is negative, only the spatially in-phase patterns are stable. When the feedback is positive, both spatially in-phase and out-of-phase patterns are stable, and the out-of-phase patterns are spatially random but satisfy that the global signals are temporal period-1 solutions.

## Introduction

Pattern formation is ubiquitous in biological systems, ranging from biological development [1, 2], ecosystems [3], to disease development [4]. Many of the pattern formation processes can be explained by Turing instability in reaction-diffusion (or activator-inhibitor) systems [5, 6]. However, pattern formation via other mechanisms has also been proposed, in particular for spatiotemporal patterns, which are also widely observed in biological systems [7–12]. The fundamental processes causing temporal and spatiotemporal dynamics in biological systems are positive and negative feedback loops [5, 6, 13]. While many studies investigated the roles of local and instantaneous feedback loops in pattern formation, studies have also carried out to investigate the roles of instantaneous global feedback and time delay global feedback (DGF) loops, such as the ones demonstrated in oscillatory media of chemical reactions [14–19]. In this study, we focus on the roles of DGF in excitation pattern formation in a class of biological systems, i.e., excitable media subjected to periodic global stimulation. Examples of pattern formation in such biological systems include subcellular calcium (Ca^2+^) release patterns in neural or cardiac cells (see examples in S1 Fig) or repolarization (voltage) patterns in cardiac tissue or cluster firings in neural networks, as described in more detail below.

**Fig 1:**
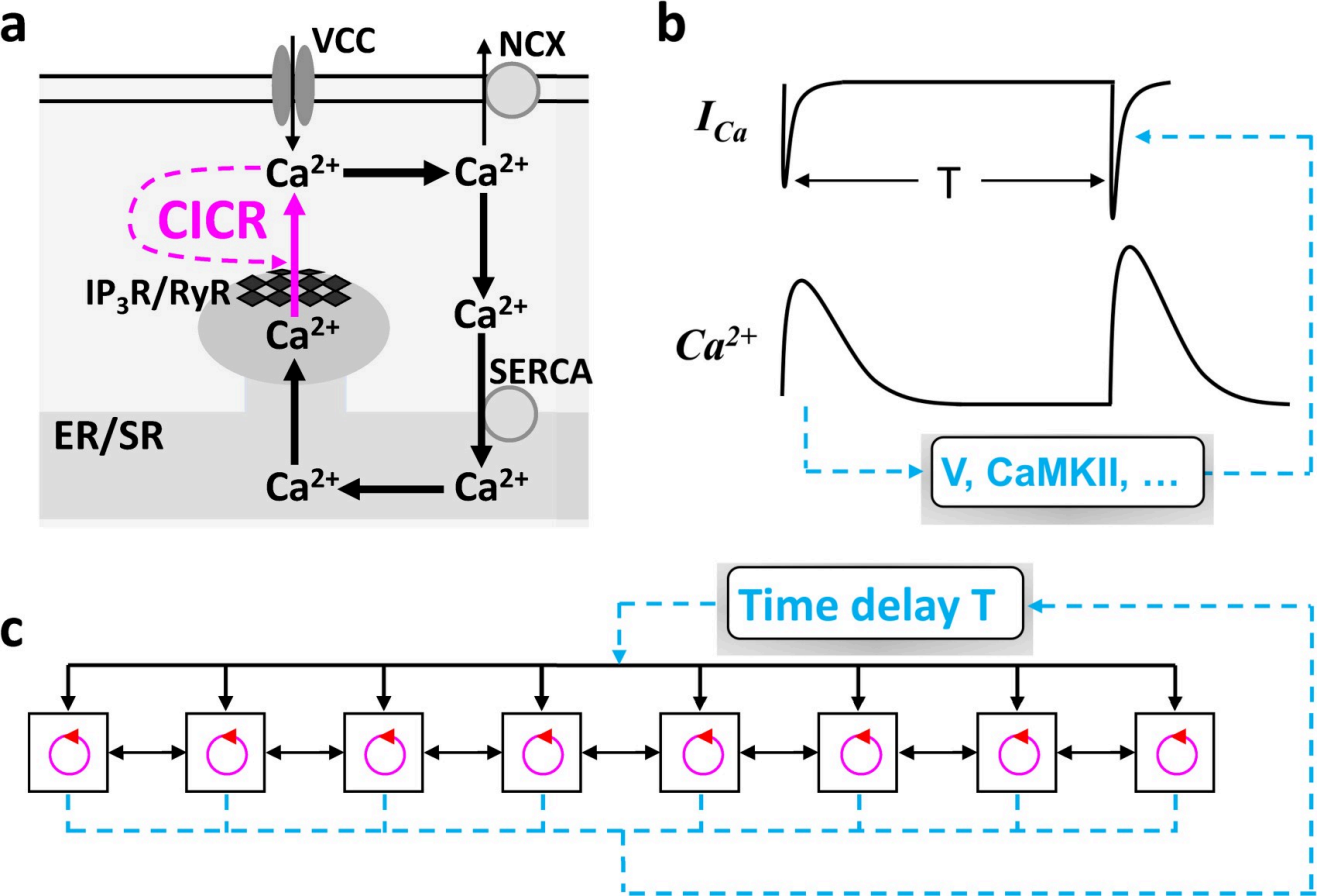
Schematic diagrams of Ca^2+^ cycling and a generic model of coupled excitable units with DGF. **a**. Schematic diagram of a basic Ca^2+^ release unit (CRU) in excitable cells. Ca^2+^ from voltage gated Ca^2+^ channels (VCCs) triggers the opening of the inositol trisphosphate receptors (IP3Rs) or ryanodine receptors (RyRs), releasing the Ca^2+^ stored in the endoplasmic or sarcoplasmic reticulum (ER/SR). The released Ca^2+^ further triggers more IP3Rs/RyRs to open, forming a positive feedback loop. This process is called Ca^2+^-induced Ca^2+^ release (CICR). Ca^2+^ is extruded by Na^+^-Ca^2+^ exchange (NCX) or other Ca^2+^ pumps and uptaken back into the ER/SR via sarco/endoplasmic reticulum Ca^2+^ ATPase (SERCA). **b**. Schematic diagram of delayed feedback in Ca^2+^ signaling via Ca^2+^ current (I_Ca_). T is the pacing period. **c**. Schematic diagram of a generic model of coupled excitable units (e.g., CRUs) with a DGF loop of time delay T.

In the chemical reaction experiments [17, 19], the DGF loops were explicitly implemented. However, the DGF loops are not obvious at all in the excitable biological systems. Here we use intracellular Ca^2+^ signaling, which is required for many biological functions [20, 21], as an example to explain the existence of DGF. The fundamental unit of Ca^2+^ signaling in a cell is called Ca^2+^ release unit (CRU) (Fig 1A). Ca^2+^ entering the cell from the voltage-gated Ca^2+^ channels triggers the opening of the Ca^2+^ release channels to release Ca^2+^ from the internal Ca^2+^ stores. The open probability of the Ca^2+^ release channels is further enhanced by the released Ca^2+^. This process is known as Ca^2+^-induced Ca^2+^ release (CICR), which is an instantaneous local feedback loop responsible for a rich spectrum of Ca^2+^ dynamics widely observed in biological systems [22–26]. Besides this instantaneous feedback loop, implicit delayed feedback loops exist, i.e., Ca^2+^ in the present beat may affect itself in the next beat (Fig 1B). This feedback can be mediated by the Ca^2+^ current (I_Ca_) of the voltage-gated Ca^2+^ channels or the Ca^2+^ release properties of the internal stores through either voltage or Ca^2+^-dependent signaling pathways. For example, in cardiac myocytes, Ca^2+^ is coupled to voltage via Ca^2+^-dependent ion channels and pumps. Changing Ca^2+^ in the present beat changes the action potential (AP) duration (APD) and thus the following diastolic interval (DI), affecting the recovery of voltage-gated Ca^2+^ channels in the next beat. As a result, the change in the recovery alters I_Ca_ and hence Ca^2+^, forming a delayed feedback loop. Note that in excitable cells, ion channels generally remain in closed or inactivation states in the quiescent phase. Therefore, the effects of this delayed feedback are manifested in the next beat. In other words, the time delay of the feedback loop is simply the pacing period T.

A cell consists of thousands of CRUs which are coupled via Ca^2+^ diffusion. The CRUs are themselves excitable units [24, 27–29], which are triggered by a global signal, i.e., voltage. Therefore, one can simplify the Ca^2+^ signaling system into a coupled array of excitable units under a global stimulation with a DGF loop (Fig 1C). Since voltage is the global signal, under normal conditions, depolarization of the cell synchronizes the firings of the CRUs, resulting in a synchronous whole-cell Ca^2+^ release, such as Ca^2+^ release in neurons (S1A Fig) [30]. The synchronous Ca^2+^ release is essential for muscle contraction [21] and many other types of biological functions [20]. However, under abnormal or diseased conditions, dyssynchronous Ca^2+^ releases can occur, such as subcellular spatially discordant Ca^2+^ alternans widely observed in cardiac myocytes (S1B Fig) [31–33]. It is clear that voltage serves as the global signal to synchronize the CRU releases, but it is unclear how dyssynchronous or discordant patterns are formed. Furthermore, the roles of the DGF in maintaining the synchronous release patterns or the development of the dyssynchronous release patterns are not well understood.

In addition to intracellular Ca^2+^ signaling, other biological systems can also be described by the simplified scheme in Fig 1C, such as the excitation dynamics in cardiac tissue or neural networks. In cardiac tissue, myocytes are electrically excitable units that are coupled via gap junctions. Contraction of the heart can serve as the global signal, which may mediate DGF via mechano-electric feedback through activating mechano-sensitive channels and affecting intracellular Ca^2+^ release [34–36]. This DGF may play essential roles in arrhythmogenic pattern formation in the heart, such as the widely observed spatially discordant APD alternans [37, 38]. In neural networks, the roles of delayed feedback in neural firing dynamics have been investigated [39–41], and DGF may also play essential roles in the formation and stability of clustered firing of neurons [42].

This study was set to investigate the roles of DGF in the genesis and stability of spatiotemporal patterns in periodically-paced biological excitable media, focusing on temporal period-2 (P2) and spatially concordant (in-phase) or discordant (out-of-phase) patterns. Mathematical models of different levels of complexity were used to allow both computer simulations and rigorous analytical treatments. First, a generic model consisting of a coupled array of excitable units described by the FitzHugh-Nagumo (FHN) model was used, and simulations were carried out to reveal the pattern dynamics caused by DGF. To validate the findings of the generic model, we used a 3-dimensional (3D) ventricular myocyte model and carried out simulations to investigate the roles of DGF in spatially concordant and discordant Ca^2+^ alternans dynamics. Of note, the term “alternans” in the context of the cardiac systems refers to a P2 state. Finally, a coupled map lattice (CML) model was used to perform detailed theoretical analyses, which provide a general mechanistic understanding of the roles of DGF in pattern formation, selection, and stability in periodically-paced biological excitable media. Our approach or objective is to use the generic FHN model to demonstrate that the DGF induced dynamics are general, use the detailed ventricular cell model to validate the general dynamics in a realistic biological system, and use the CML model to reveal the underlying dynamical mechanisms. We argue (as detailed in Discussion) that besides the subcellular spatiotemporal Ca^2+^ dynamics in ventricular myocytes, due to the generality of the simple models, the mechanistic insights shall also be applicable to spatially discordant APD alternans in cardiac tissue or cluster firings in neural networks when global feedback or DGF exists. The theoretical insights may also be helpful for understanding the mechanisms of pattern formation induced by global feedback or DGF in oscillatory chemical media shown in previous studies [14–19].

## Results

### DGF in the genesis of spatiotemporal dynamics in an array of coupled FHN units

We used a generic model consisting of a one-dimensional (1D) array of coupled FHN units to investigate the spatiotemporal excitation patterns. The governing differential equations are:

$$\begin{array}{c} \frac{dc(j,t)}{dt}=f[c(j,t),w(j,t)]+D[c(j+1,t)+c(j-1,t)-2c(j,t)]+I(t)\\ \frac{dw(j,t)}{dt}=g[c(j,t),w(j,t)] \end{array}$$ (1)

in which *j = 1*, *2*, *…*, *L* is the spatial index of the FHN units with *L* being the length of the 1D array. We used the standard FHN kinetics, i.e.,

$$f(c,w)=-c(c-1)(c-c_{th})-0.1w,g(c,w)=(c-0.25w-0.3)/10$$ (2)

where *c* is the activator and *w* the inhibitor. *c*_*th*_ = 0.5 is a parameter determining the threshold for excitation, and *D* = 0.1 is the coupling strength. *I(t)* in Eq 1 is the external stimulus pulse. No-flux boundary condition was used.

As implied in Fig 1B, I_Ca_ that triggers the Ca^2+^ release and mediates the feedback is a spiky current, which only occurs at the beginning of each pacing beat. Therefore, instead of adding a time-continuous feedback term to Eq 1, we add the feedback to the stimulus pulse, which is then formulated as

$$I(t)=\left\{ \begin{array}{c} I_{0}[1+\alpha ({\bar{c}}_{n-1}-{\bar{c}}_{s})],if\;nT<t<nT+\Delta T\\ 0,otherwise \end{array}\right.$$ (3)

In Eq 3, *n* is the index of the beat number, *T* is the pacing period, Δ*T* is the pulse duration, and *α* is the feedback strength. ${\bar{c}}_{n-1}$ is the peak value of the spatial average of *c* (denoted as $\bar{c}(t)$) at the (n-1)^th^ beat. ${\bar{c}}_{s}$ is the reference value for the feedback. Here we define *α*>0 as positive feedback (*α*<0 as negative feedback), since in an uncoupled FHN unit, a larger *c*_*n*−1_ gives rise to a larger *I*(*t*), and thus a larger *c*_*n*_. We set *I*_0_ = 1.2, Δ*T* = 0.5, and ${\bar{c}}_{s}=0.77$. The ${\bar{c}}_{s}$ value is roughly the steady-state value of c at the bifurcation point from P1 to P2 of an FHN unit without feedback. We find that other choices do not change the general results but exhibit certain quantitative differences.

Random initial conditions are used to induce spatially discordant patterns. Specifically, at t = 0, we set the variable *w* to be a spatially random distribution, i.e., *w*(*j*,0) = 0.5+*δξ*(*j*) (j = 1,2,…,L) with *ξ*(*j*) being a uniform random number in [–1,1]. δ is the parameter determining the extent of the spatial heterogeneity and the initial condition becomes uniform when *δ* = 0.

#### Excitation patterns without DGF

**Fig 2:**
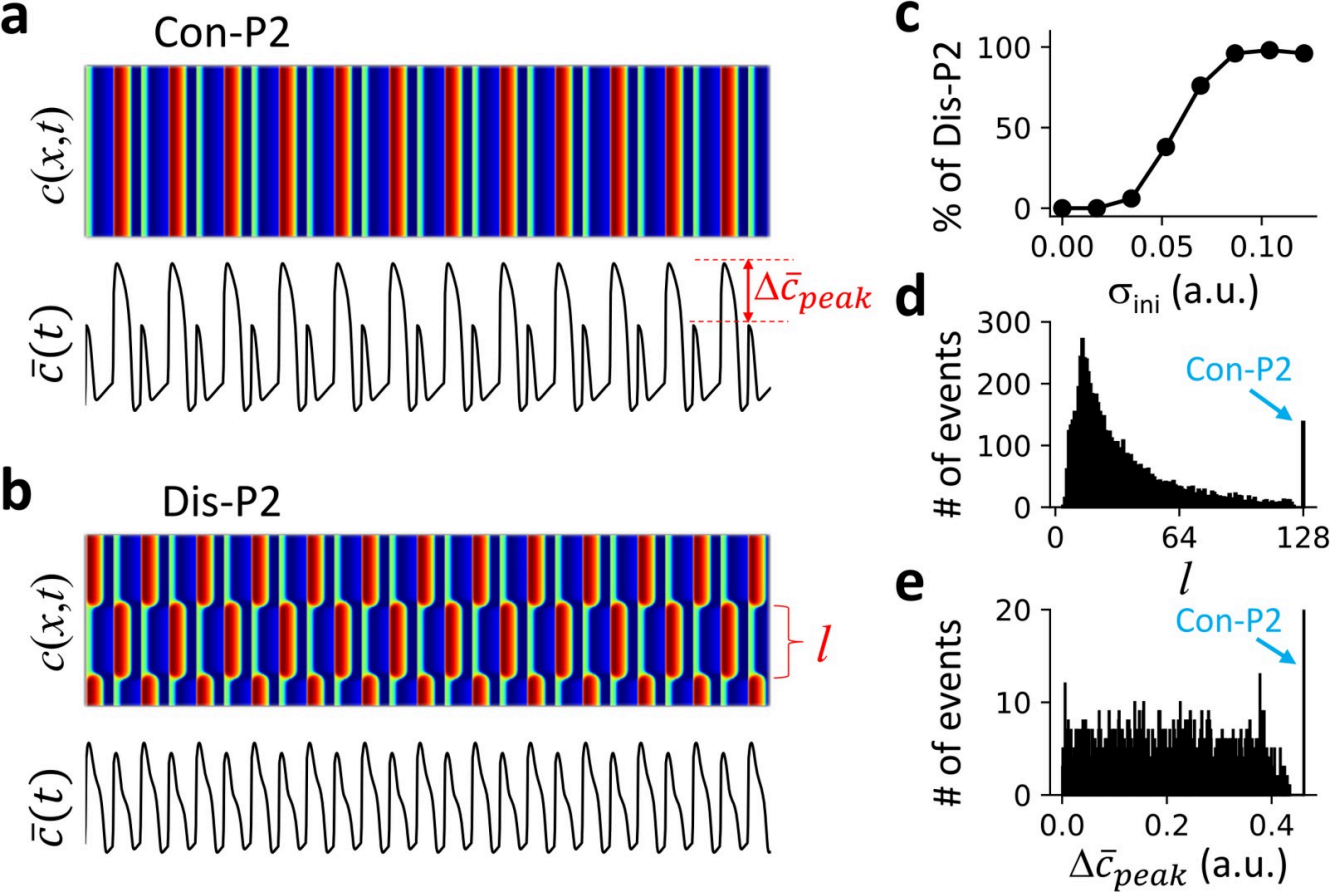
Excitation patterns and dynamics in a 1D array of coupled FHN units without DGF. The pacing period T = 45 and system size L = 128. **a**. An example of Con-P2 patterns and the corresponding global signal $\bar{c}(t)$. **b**. An example of Dis-P2 patterns with a different initial condition from panel a, and the corresponding $\bar{c}(t)$. **c**. Percentage of Dis-P2 patterns versus the standard deviation (*σ*_*ini*_ of the random initial conditions. $\sigma_{ini}=\delta /\sqrt{3}$. We performed 100 trials for each *σ*_*ini*_ value in the plot. **d**. Histogram of domain size *l* (segment between two neighboring nodes, as indicated in panel b) from 2000 trials of random initial conditions with *δ* = 0.15. For each trial, 2000 beats were applied for the system to reach a steady state. The domain size was measured using the last two beats. **e**. Histogram of global P2 amplitude $\Delta {\bar{c}}_{peak}$, (difference between the peak values of two consecutive beats, as indicated in panel a) for the simulations in d. $\Delta {\bar{c}}_{peak}$ was measured using the last two beats.

In the absence of DGF, i.e., *α* = 0 in Eq 3, a bifurcation from temporal P1 to P2 occurs as the pacing period T decreases in an uncoupled FHN unit (S2 Fig). We use spatially random initial conditions as detailed above to induce discordant patterns in the 1D array. We find that when T first passes through the bifurcation point, the system can only exhibit a spatially concordant P2 (Con-P2) pattern independent of initial conditions. As T decreases further, the system can exhibit a Con-P2 (Fig 2A) or a spatially discordant P2 (Dis-P2) pattern (Fig 2B) depending on the initial condition. The probability of forming a Dis-P2 pattern increases as the spatial heterogeneity of the initial condition increases (Fig 2C). Moreover, the Dis-P2 patterns are spatially random and selected by initial conditions. To quantify this property, we measured the spatial domain sizes (see Fig 2B for definition) from 2000 random trials for a given standard deviation of the spatial heterogeneity of the initial condition, and plotted the corresponding histogram in Fig 2D. It shows that the domain size can be any value as long as it is greater than a minimum domain size *l*_min_, i.e., the domain sizes distribute between *l*_min_ and *L*-*l*_min_. Because of this randomness in pattern selection, the corresponding histogram of the global P2 amplitude ($\Delta {\bar{c}}_{peak}$ as defined in Fig 2A) also exhibits a continuous distribution (Fig 2E).

#### Effects of DGF on pattern selection and stability

**Fig 3:**
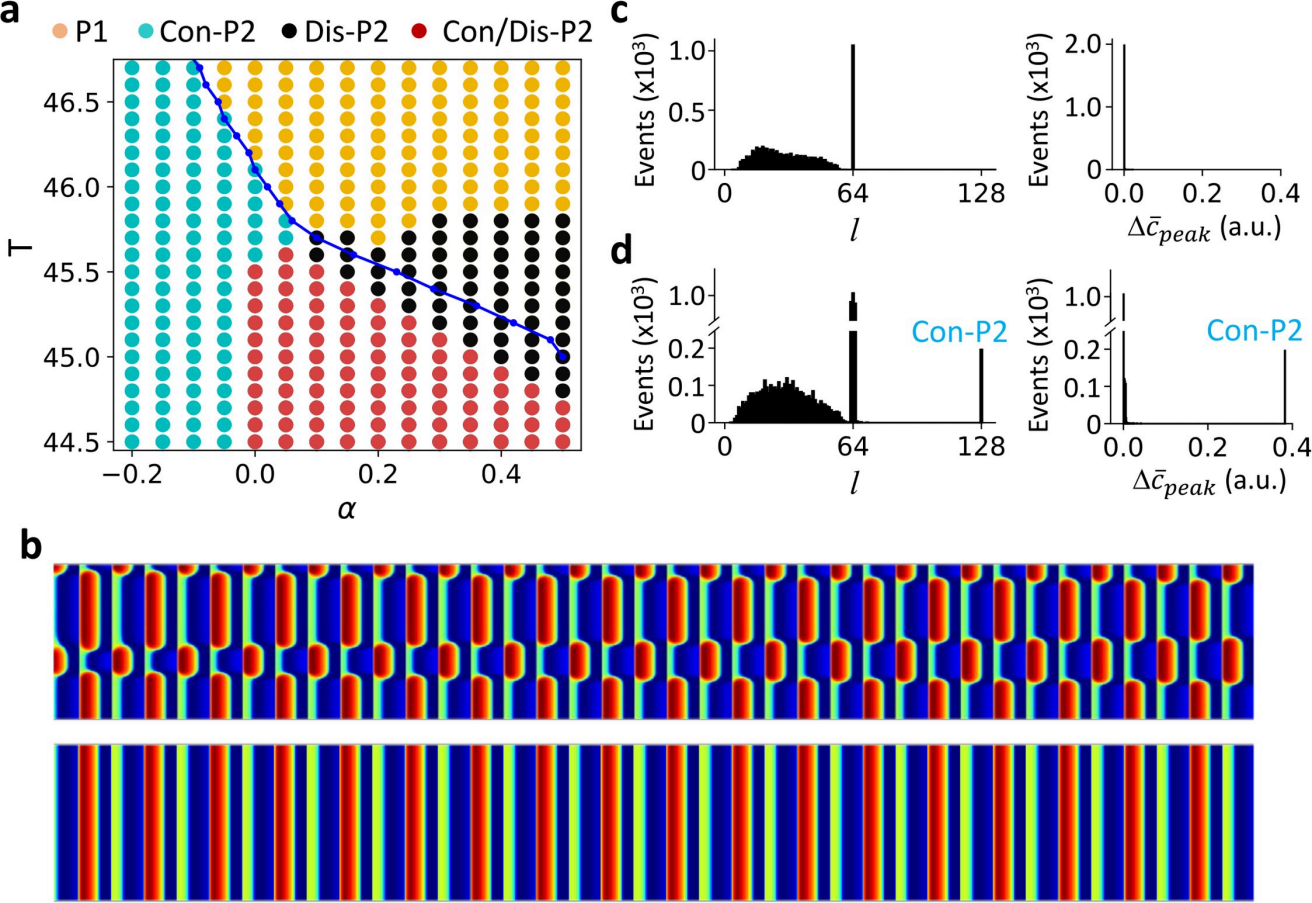
Excitation patterns and dynamics in a 1D array of coupled FHN units with DGF. **a**. Phase diagram of excitation dynamics. The blue line is the bifurcation boundary from P1 to P2 in an uncoupled unit with DGF. Color dots mark the different behaviors in the 1D array: yellow—uniform P1; black—Dis-P2; cyan—Con-P2; and red—Con/Dis-P2. **b**. A Dis-P2 pattern (upper) and a Con = P2 pattern (lower) for *α* = 0.2 and T = 45 obtained with two different initial conditions. **c**. Left, histogram of domain size *l* from 2000 trials. α = 0.4 and T = 45.5. The random initial conditions were set the same way as described in Fig 2 legend with δ = 3. For each trial, 2000 beats were applied for the system to reach a steady state. Right, corresponding histogram of global P2 amplitude $\Delta {\bar{c}}_{peak}$ from the same simulations. **d**. Same as panel c but for α = 0.2, T = 45, and δ = 0.09.

To investigate the effects of DGF on the spatiotemporal pattern dynamics, we carried out simulations by scanning the pacing period T and DGF strength α (Fig 3A). There are four distinct regions: uniform P1 pattern (yellow), Con-P2 pattern only (cyan), Dis-P2 pattern only (black), and both concordant and discordant P2 (Con/Dis-P2) patterns (red). The blue curve is the stability boundary between P1 and P2 for an uncoupled FHN unit. For *α*<0, only uniform P1 and Con-P2 patterns were observed, independent of initial conditions. The uniform P1 and Con-P2 patterns are separated by the stability boundary (blue line) of the uncoupled FHN unit, indicating that the dynamics in the 1D array is the same as in the uncoupled FHN unit. For *α*>0, a transition from uniform P1 to Dis-P2 occurs as T decreases (from yellow to black), which is caused by a spatial-mode instability of the uniform P1 state. As T decreases further (red region), both Con-P2 and Dis-P2 patterns can occur depending on the initial conditions (Fig 3B).

Furthermore, we performed the same statistical analysis as in the case of no DGF (Fig 2D and 2E) for different regions of the phase diagram in Fig 3A. In the Dis-P2 only region (Fig 3C), the domain sizes distribute between 0 to *L*/2 (more accurately, the domain size can be *L*/2 and any value between *l*_min_ and *L*/2-*l*_min_), but $\Delta {\bar{c}}_{peak}$ remains zero for all patterns. In the Con/Dis-P2 region (Fig 3D), the distributions are similar to those in Fig 3C except for the existence of the Con-P2 pattern. Similar to the case of no DGF (*α* = 0), the domain size distributions are continuous, indicating that the Dis-P2 patterns are spatially random (including the periodic ones) and depend on initial conditions. However, differing from the case of no DGF, the global signals of the Dis-P2 patterns are always P1 solutions i.e., $\Delta {\bar{c}}_{peak}=0$ (Fig 3C and 3D). Moreover, the maximum domain size of Dis-P2 patterns is *L*/2. This is because if there is a domain greater than *L*/2, the sum of all other domains must be smaller than *L*/2, and thus when the patterns reverse in the next beat, the global signal cannot be the same, violating the requirement of a global P1 solution.

Therefore, in the absence of DGF (*α* = 0), both Con-P2 and Dis-P2 patterns can occur, and the Dis-P2 patterns are spatially random. In the presence of DGF, only Con-P2 patterns can exist when the DGF is negative (*α*<0). When the DGF is positive (*α*>0), both Con-P2 and Dis-P2 patterns can exist depending on the pacing period T and initial conditions. The Dis-P2 patterns are also spatially random but satisfy that the global signals are always P1 solutions.

### Ca^2+^ release patterns in a physiologically detailed ventricular myocyte model

To validate the spatiotemporal dynamics in a realistic biological system, we carried out simulations in a physiologically detailed 3D ventricular myocyte model (see Methods), which can well capture the spatiotemporal Ca^2+^ dynamics widely observed in experiments [29, 43–45]. The model undergoes a bifurcation from P1 to P2 (alternans) as the pacing period T decreases (S2 Fig). We investigated the subcellular Ca^2+^ release patterns under both AP clamp and free-running conditions (see S1 Text). Under AP clamp, the voltage is no longer described by the differential equation but a pre-set function of time with a period T (see S3 Fig for the waveform used in this study). Under this condition, Ca^2+^ is decoupled with voltage, and therefore, there is no DGF. Under the free-running condition, the cell is stimulated by a current pulse (0.5 ms duration and -80 μA/μF amplitude, see Methods), and voltage is a free running signal. Under this condition, Ca^2+^ and voltage are coupled, and therefore, DGF is present due to Ca^2+^ and voltage coupling (as indicated in Fig 1B and 1C). The DGF properties can be changed by altering the ionic currents, as will be detailed below.

#### Ca^2+^ release patterns under AP clamp

**Fig 4:**
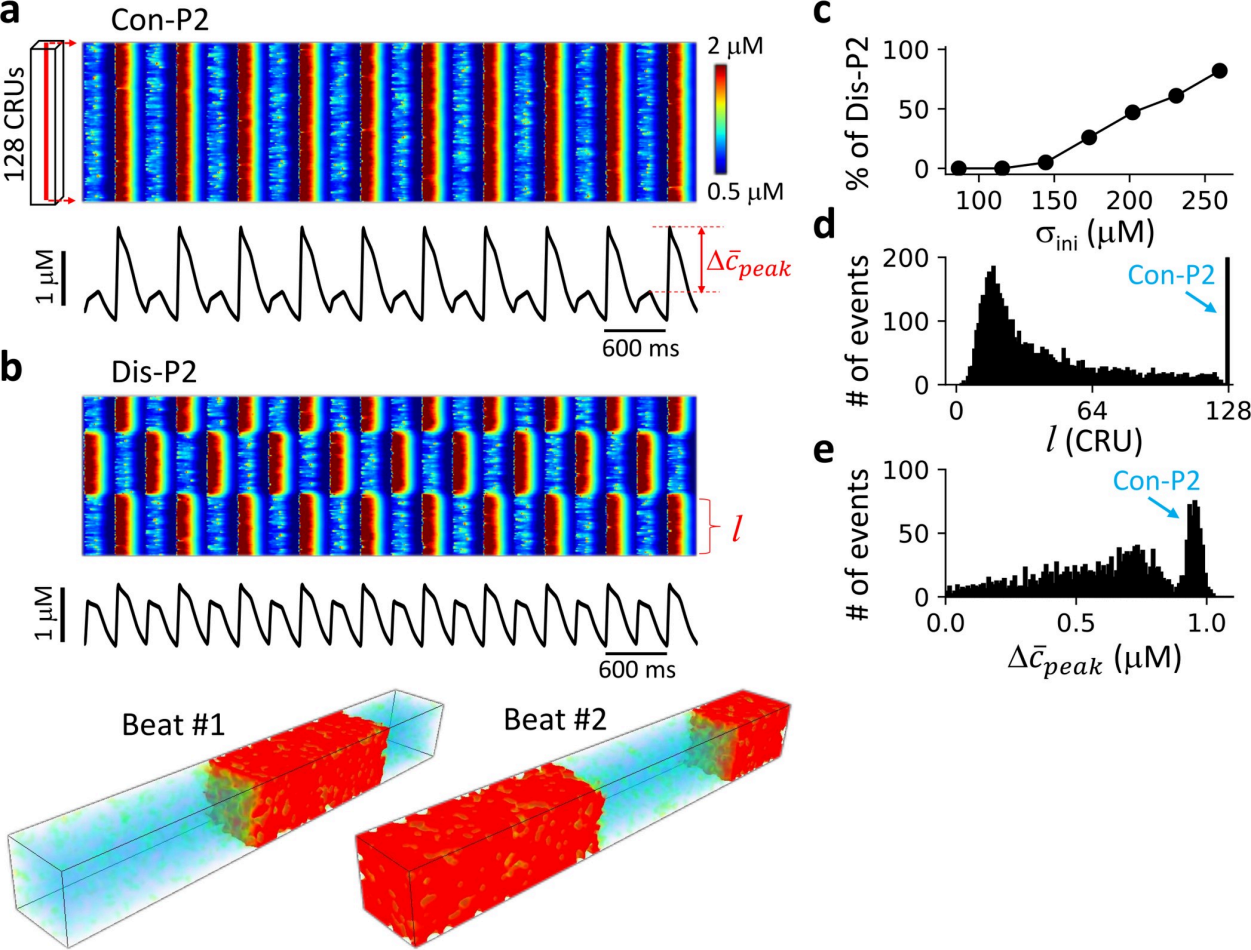
Ca^2+^ release patterns and dynamics in the 3D ventricular myocyte model under AP clamp. **a**. Upper panel shows a linescan (time-space plot) of cytosolic Ca^2+^ concentration showing a Con-P2 pattern. Lower panel shows the corresponding whole-cell Ca^2+^ transient. The recording line was in the center of the cell as indicated on the left. **b**. Same as panel a. with a different random initial condition resulting in a Dis-P2 pattern. The middle panel is the corresponding whole-cell Ca^2+^ transient. The bottom panels are 3D views of Ca^2+^ from two consecutive beats. **c**. Percentage of Dis-P2 patterns versus the standard deviation (*σ*_*ini*_)of initial SR Ca^2+^ load. The random spatial distribution of the SR Ca^2*+*^ load was set as *Ca*_*SR*_(*j*) = *Ca*_0_+Δ*Ca*_*SR*_∙*ξ*(*j*)·(j = 1,2,…,L) with *ξ*(*j*) being a uniform random number in [–1,1]. $\sigma_{ini}=\Delta Ca_{SR}/\sqrt{3}$. We set *Ca*_0_ = 500 μM. We performed 100 trials for each *σ*_*ini*_ value in the plot. **d**. Histogram of domain size *l* (as marked in panel b) with Δ*Ca*_*SR*_ = 450 μM. **e**. Histogram of global P2 amplitude $\Delta {\bar{c}}_{peak}$ (as marked in panel a) from the same simulations in panel d. For panel d and e, 2000 trials were performed. For each trial, the cell was paced 2000 beats to reach a steady state. The domain size was computed using the last 50 beats to account for beat-to-beat variation (see SI for details) due to the intrinsic noise of ion channel stochasticity. $\Delta {\bar{c}}_{peak}$ was measured using the last two beats. The pacing period T = 300 ms.

Under AP clamp, Ca^2+^ is decoupled with voltage and thus no DGF. We used spatially random initial conditions to induce Dis-P2 patterns (see Fig 4 legend for the details of the random initial conditions). In the alternans regime (e.g., T = 300 ms), both Con-P2 (Fig 4A) and Dis-P2 (Fig 4B) patterns occur in the cell depending on initial conditions. The probability of forming a Dis-P2 pattern increases as the spatial heterogeneity of initial conditions increases (Fig 4C). The Dis-P2 patterns are spatially random as indicated by the histograms of domain size (Fig 4D) and whole-cell alternans amplitude $\Delta {\bar{c}}_{peak}$ (Fig 4E). These behaviors are the same as those for the model of coupled FHN units without DGF (Fig 2), albeit some smearing in the histograms due to ion channel stochasticity (see Methods).

#### Ca^2+^ release pattern dynamics with positive and negative Ca^2+^-to-APD coupling

Under free running, however, Ca^2+^ is coupled with voltage, and changing Ca^2+^ may change APD. If increasing the Ca^2+^ transient amplitude results in a longer APD in the same beat, then it is called positive Ca^2+^-to-APD coupling, and the opposite is called negative Ca^2+^-to-APD coupling [46, 47]. As shown in previous studies [46, 48, 49], the coupling properties can be altered by altering the properties of I_Ca_ and the Na^+^-Ca^2+^ exchange current (I_NCX_). However, since I_Ca_ and I_NCX_ also directly affect the Ca^2+^ dynamics, it becomes difficult to only change the Ca^2+^-to-APD coupling without affecting the Ca^2+^ dynamics. To avoid this complexity, we added two new currents to the ventricular model: the non-specific Ca^2+^-activated cation current (I_nsCa_) and the small conductance Ca^2+^-activated potassium current (I_SK_). I_nsCa_ is mainly an inward current [50] and increases as the intracellular Ca^2+^ concentration increases. Therefore, increasing the conductance of I_nsCa_ increases APD and promotes positive Ca^2+^-to-APD coupling. I_nsCa_ is small under normal conditions but becomes large under conditions of Ca^2+^ overload [50]. I_SK_ is an outward current which also increases with the intracellular Ca^2+^ concentration, and thus I_SK_ promotes negative Ca^2+^-to-APD coupling. Under normal conditions, I_SK_ only presents in atrial myocytes, but under heart failure, it also presents in ventricular myocytes [51–53]. Since the charge carriers of both currents are not Ca^2+^, unlike I_Ca_ and I_NCX_, they do not directly affect intracellular Ca^2+^ dynamics. Here we altered the maximum conductance of these two currents to alter the Ca^2+^-to-APD coupling properties. Note that DGF is not explicitly present in the model but indirectly via the Ca^2+^-to-APD coupling, as we explained in Introduction. We first investigated the effects of Ca^2+^-to-APD coupling properties on Ca^2+^ release patterns and then linked them to DGF.

**Fig 5:**
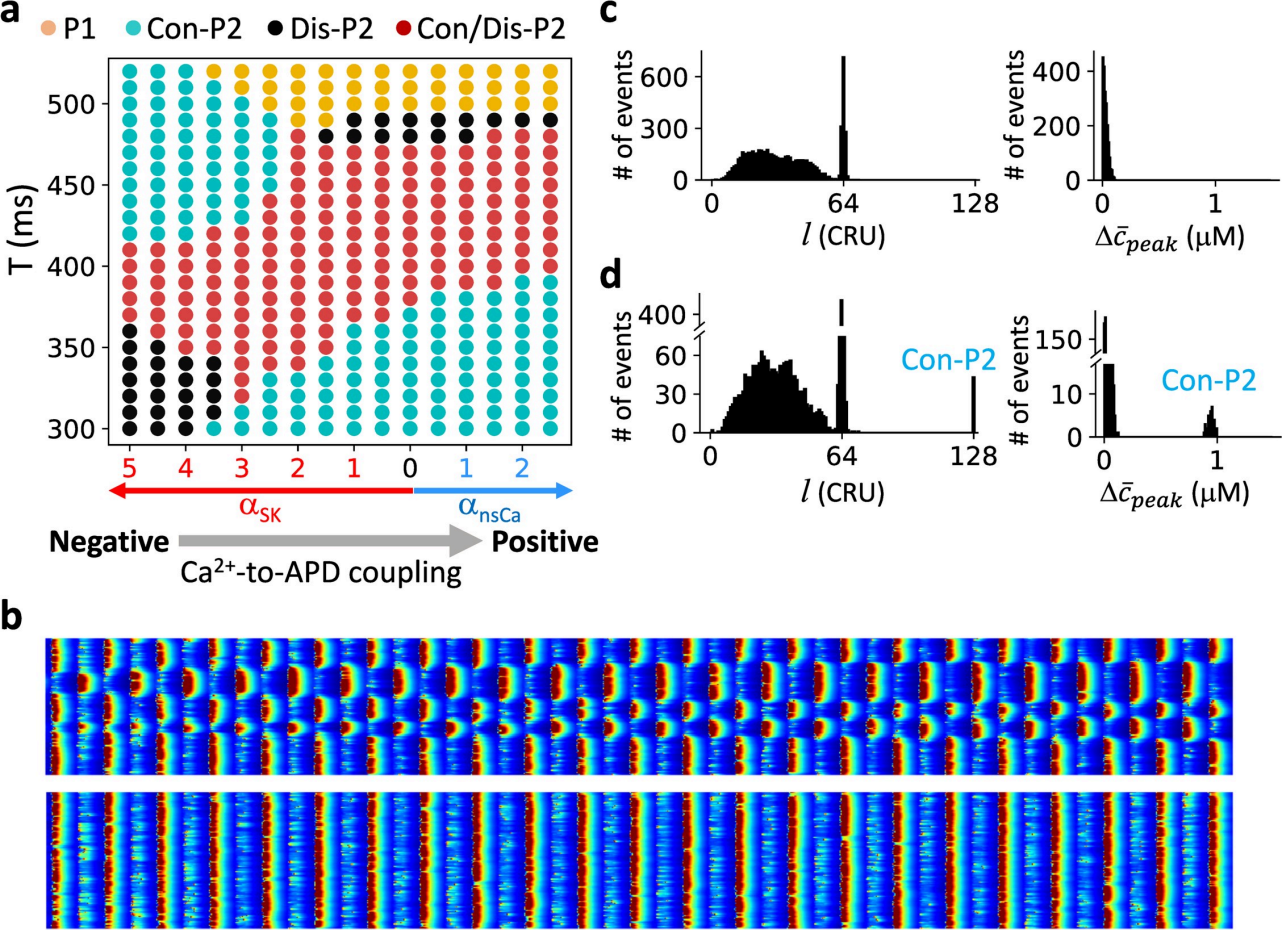
Ca^2+^ release pattern dynamics in the ventricular myocyte model with positive and negative Ca^2+^-to-APD coupling. **a**. Phase diagram of Ca^2+^ release dynamics versus pacing period and Ca^2+^-to-APD coupling properties. In this diagram, the x-axis is the fold increase of either *I*_nsCa_ (blue arrow) or *I*_SK_ (red arrow), and the y-axis is the pacing period T. Gray arrow indicates the change from negative to positive Ca^2+^-to-APD coupling. Same color codes of the pattern dynamics as in Fig 3A were used. **b**. A Dis-P2 pattern (upper) and a Con = P2 pattern (lower) for *α*_*SK*_ = 3.5 and T = 350 ms obtained with two different initial conditions. **c**. Left: Histogram of domain size *l*. The pacing period T = 330 ms, *α*_*SK*_ = 4.5, The random initial conditions were set the same way as in Fig 4 with Δ*Ca*_*SR*_ = 500 μM. Two thousand trials were performed. For each trial, the cell was paced 2000 beats to reach a steady state. The domain size was computed using the last 50 beats. Right: Histogram of global P2 amplitude $\Delta {\bar{c}}_{peak}$ from the same simulations. $\Delta {\bar{c}}_{peak}$ was measured using the last two beats. **d**. Same as panel c but T = 350 ms and *α*_*SK*_ = 3.5.

We systematically explored the spatiotemporal dynamics by altering the pacing period T and the maximum conductance of the two currents, as summarized in Fig 5A. We used the same spatially random initial conditions as in Fig 4 for the simulations. Note that due to dynamical instabilities and intrinsic noise of the sarcolemmal Ca^2+^ channels and Ca^2+^ release channels (see Methods), heterogeneous initial conditions are not needed for the formation of the Dis-P2 patterns in the Dis-P2 only region. When the Ca^2+^-to-APD coupling is negative (large I_SK_), a transition from uniform P1 to Con-P2 patterns occurs as T decreases, and this transition occurs at a larger T value as the maximum I_SK_ conductance increases. When the coupling is positive (large I_nsCa_), a transition from uniform P1 to Dis-P2 patterns (yellow to black) occurs as T decreases. Under both coupling conditions, as T decreases further, the system enters the Con/Dis-P2 regime (red), in which both Con-P2 and Dis-P2 patterns can occur depending on initial conditions (Fig 5B). However, as T decreases even further, the Con/Dis-P2 regime switches into a Dis-P2 only regime when the Ca^2+^-to-APD coupling is negative (large I_SK_) and into a Con-P2 only region when the Ca^2+^-to-APD coupling is positive (large I_nsCa_). Therefore, for the same Ca^2+^-to-APD coupling, as T decreases, the spatiotemporal patterns change from Con-P2 only to Dis-P2 only through a Con/Dis-P2 region or in reverse order depending on the coupling properties.

To reveal the statistical properties of the Dis-P2 patterns, we show the histograms of domain sizes and the whole-cell alternans amplitude for a parameter point in the Dis-P2 region (Fig 5C) and a point in the Con/Dis-P2 region (Fig 5D). The domain size distributions for Dis-P2 patterns are continuous, and the whole-cell alternans amplitudes always remain zero, indicating that the patterns are spatially random but always satisfy that the global signals are P1 solutions. These behaviors are the same as in the model of a coupled array of FHN units (Fig 3).

#### Linking the Ca^2+^-to-APD coupling properties to DGF

**Fig 6:**
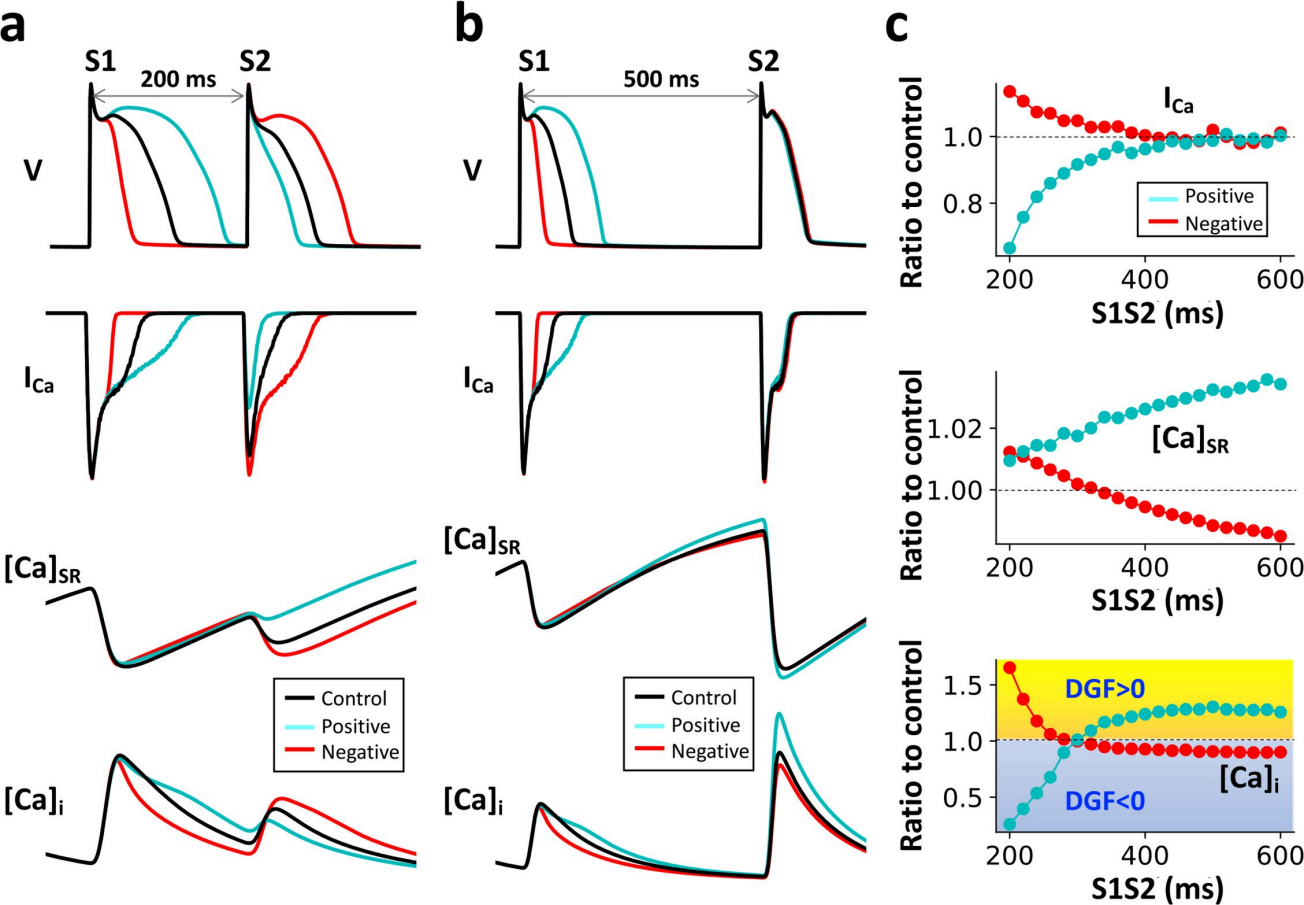
Relationship between the Ca^2+^-to-APD coupling properties and DGF. **a.** Time traces of voltage, I_Ca_, SR Ca^2+^, and cytosolic Ca^2+^ for control (black), enhanced positive coupling in the S1 beat (cyan) and enhanced negative coupling in the S1 beat (red). S1S2 = 200 ms. **b.** Same as in a. but S1S2 = 500 ms. **c.** Ratios of I_Ca_ peak, SR Ca^2+^ load, and [Ca^2+^]_i_ peak on the S2 beat under positive (cyan) or negative (red) coupling conditions to the corresponding quantities at control v.s. S1S2 interval. Here, *α*_*nsCa*_ = 0 for all the cases. In the S1 beat, *α*_*SK*_ is 5, 0, and 30 for control, enhanced positive, and enhanced negative cases, respectively. In the S2 beat, *α*_*SK*_ is 5 for all the cases.

As shown above, the Ca^2+^ dynamics in the detailed ventricular myocyte model agree well with those of the generic FHN array with DGF. To link the spatiotemporal Ca^2+^ dynamics to the DGF properties, we performed a quantitative analysis to reveal the DGF properties and their relationship with the Ca^2+^-to-APD coupling properties. We carried out simulations of the ventricular cell model using an S1S2 protocol. In this protocol, a train of external stimuli (S1) was given at a fixed period (T = 300 ms) for the cell to reach the steady state, and a stimulus (S2) was then applied after a variable interval (denoted as S1S2 interval) from the last S1 beat. We measured the peak values of I_Ca_, SR Ca^2+^ and cytosolic Ca^2+^ on the S2 beat, which were considered as control. Next, we repeated the same simulations except that on the last S1 beat, we increased α_SK_ to shorten the APD (Fig 6A, red) or increased α_nsCa_ to lengthen the APD (Fig 6A, cyan). This method enhanced the negative (increased α_SK_) or the positive (increased α_nsCa_) Ca^2+^-to-APD coupling from the control case. In Fig 6C, we plot the ratios of the peak values of I_Ca_, SR Ca^2+^ and cytosolic Ca^2+^ of the two coupling cases to the control values on the S2 beat.

For short S1S2 intervals (e.g., S1S2 = 200 ms, Fig 6A), the enhanced positive Ca^2+^-to-APD coupling case (cyan) results in a smaller I_Ca_ (ratio <1, see Fig 6C, top panel) and almost unchanged SR load compared to those of the control case on the S2 beat. The reason is that the longer APD caused by the positive Ca^2+^-to-APD coupling results in a shorter DI and thus less recovery of I_Ca_. The net effect is to result in a smaller Ca^2+^ transient (ratio < 1, see Fig 6C, bottom panel) than that of the control case because of a smaller I_Ca_. This indicates that the global feedback results in a negative gain on the Ca^2+^ signal on the next beat, i.e., the DGF is negative. For the negative Ca^2+^-to-APD coupling case (Fig 6A, red), the consequences are reversed, resulting in a positive DGF (Fig 6C, bottom panel). These results suggest that for short S1S2 intervals, the DGF properties are primarily determined by the recovery property of I_Ca_.

For long S1S2 intervals (e.g., S1S2 = 500 ms, Fig 6B), the Ca^2+^-to-APD coupling properties have negligible effects on I_Ca_ (both ratios are ~1, see Fig 6C, top panel) since I_Ca_ fully recovers due to a long DI. However, the SR Ca^2+^ load is lower (ratio<1) for the negative Ca^2+^-to-APD coupling and higher (ratio>1) for the positive Ca^2+^-to-APD coupling (Fig 6C, middle panel). Therefore, for the negative Ca^2+^-to-APD coupling case, the feedback results in a smaller Ca^2+^ in the next beat because of a lower SR Ca^2+^ load, i.e., the DGF is negative. For the positive Ca^2+^-to-APD coupling case, the DGF is positive. These results suggest that for long S1S2 intervals, the DGF properties are primarily determined by the SR Ca^2+^ load.

Therefore, for short S1S2 intervals (<300 ms), positive or negative Ca^2+^-to-APD coupling leads to negative or positive DGF, respectively, primarily mediated by I_Ca_ recovery. For long S1S2 intervals (>300 ms), the relationships are reversed, i.e., positive or negative Ca^2+^-to-APD coupling leads to positive or negative DGF, respectively. The DGF is primarily mediated by SR Ca^2+^ load since with long S1S2 intervals the Ca^2+^-to-APD coupling properties show almost no effects on I_Ca_ due to full recovery. Knowing the relationships between Ca^2+^-to-APD coupling properties and the DGF properties, one can then link the DGF properties to the Ca^2+^ release patterns in the ventricular cell model. Based on the analysis above and the Ca^2+^ release patterns shown in Fig 5A, we can link the Ca^2+^ release patterns to the sign of DGF, which is summarized in Table 1. It shows that negative DGF leads to Con-P2 patterns, while positive DGF leads to Dis-P2 patterns. Note that we were not able to quantitatively distinguish the boundaries between positive and negative DGF in the phase diagram (Fig 5A) using this analysis, but it is likely that in the Con/Dis-P2 region, the DGF is also positive. Therefore, we can obtain the same conclusion as that from the generic FHN model.

**Table 1:** Links between Ca^2+^-to-APD coupling, Ca^2+^ release patterns, and DGF for the detailed ventricular cell model. The sign in parenthesis is the sign of DGF.

	Negative Ca^2+^-to-APD coupling	Positive Ca^2+^-to-APD coupling
Slow pacing	Con-P2, DGF (-)	Dis-P2, DGF (+)
Fast pacing	Dis-P2, DGF (+)	Con-P2, DGF (-)

### Theoretical insights from a CML model

To reveal analytically the instabilities and bifurcations leading to the spatiotemporal dynamics, we used a CML model to describe the system. CML, as a generic model for investigating spatiotemporal dynamics of nonlinear systems, has been widely used [54, 55]. In a previous study [56], we developed a CML model to investigate the spatiotemporal APD dynamics in cardiac tissue. Here we modified the 1D array CML model by adding a DGF term. The governing equation is,

$$c_{n}(j)=f[c_{n-1}(j)]+\sum_{m=-M}^{M}w_{m}[f[c_{n-1}(j+m)]-f[c_{n-1}(j)]]-\alpha [f({\bar{c}}_{n-1})-f({\bar{c}}_{s})]$$ (4)

where *n* is the temporal index and *j* the spatial index. *c*_*n*_(*j*) describes the peak signal in the *j*^th^ lattice of the *n*^th^ beat. *f* is the map function: $f(c_{n})=0.2+\frac{0.8}{1+e^{(c_{n}-\gamma )/\mu }}$, in which γ and *μ* determine the midpoint and the slope of the curve, respectively. M is the coupling length, and w_m_ is the coupling strength described by a Gaussian function: $w_{m}=\frac{e^{-m^{2}/2\sigma^{2}}}{\sqrt{2\pi }\sigma N_{w}}$ in which *N*_*w*_ is the normalization constant. ${\bar{c}}_{n-1}$ is the spatial average of *c*_*n*−1_(*j*), and ${\bar{c}}_{s}=c_{s}$ satisfying *c*_*s*_ = *f*(*c*_*s*_). We set *μ* = 0.1, M = 15, and *σ* = 3. Note that concrete forms of map functions for investigating intracellular Ca^2+^ dynamics were developed previously [47, 57]. However, for the sake of simplicity and generality, we chose a simple function *f*, which can give rise to a bifurcation from P1 to P2, similar to those of the FHN model and the detailed ventricular myocyte model (see S2 Fig).

Note that in Eq 4, instead of using a linear feedback term: $\alpha ({\bar{c}}_{n-1}-{\bar{c}}_{s})$, we used a nonlinear term with the map function *f*, i.e., $-\alpha [f({\bar{c}}_{n-1})-f({\bar{c}}_{s})]$, to maintain the convergence of iteration. The negative sign was used because *f* is a decreasing function (*f*′<0). Linearization of this nonlinear term around the uniform steady-state gives rise to a term proportional to $\alpha ({\bar{c}}_{n-1}-{\bar{c}}_{s})$, and thus *α*>0 corresponds to positive feedback, the same as in the FHN model.

*1) Stability of an uncoupled unit*. For an uncoupled unit, the map equation with DGF becomes

$$c_{n}=f(c_{n-1})-\alpha [f(c_{n-1})-f(c_{s})]$$ (5)

**Fig 7:**
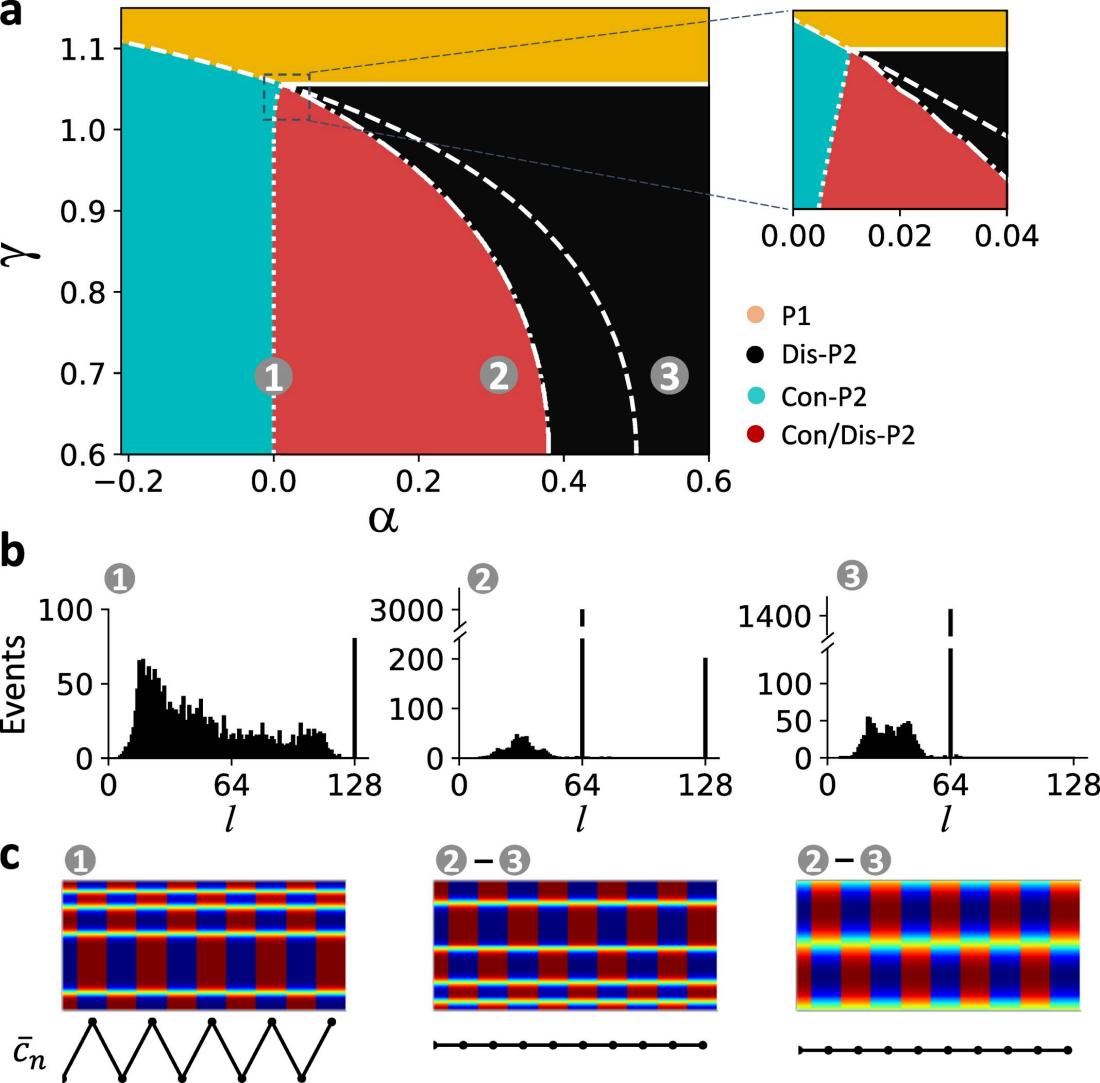
Bifurcations and spatiotemporal dynamics in the CML model. **a**. Phase diagram in the α-γ space showing stability boundaries and spatiotemporal dynamics of the CML model. The same color codes as in Fig 3A and Fig 5A were used. The solid line is the stability boundary of uniform P1 determined by Eq 7. The dashed line is the stability boundary of P1 in an uncoupled unit determined by Eq 6. The dash-dot line is the stability boundary of Con-P2 determined by Eq 8. The vertical dotted line is the stability boundary of Dis-P2 determined by numerical simulations of the CML model. Inset is the blowup of the marked region, showing that all the stability boundaries meet at a common point. **b**. Histograms of domain size at different locations in the phase diagram marked by numbers. The coordinates (α, γ) from location 1 to 3 are: (0, 0.7), (0.3, 0.7), and (0.55, 0.7). **c**. Sample spatiotemporal patterns (top) and the corresponding global signals (bottom) from different regions of the phase diagram. Number ranges above each pattern indicate the locations in the phase diagram where the specific pattern can be seen.

The stability of the steady-state solution is determined by the eigenvalue,

λ=(1−α)f′ (6)

where $f^{\prime }={\frac{df}{dc_{n}}|}_{c_{n}=c_{s}}.\;f^{\prime }$ is independent of *α* since the steady state is independent of *α*. Eq 6 indicates that *α*<0 destabilizes the steady state, and *α*>0 stabilizes the steady state. The stability boundary is shown as the dashed line in Fig 7A.

*2) Stability of the spatially uniform P1 state*. The spatially uniform P1 state (see Stability analyses of the CML model in SI) is determined by the eigenvalues:

$$\lambda_{k}=\left\{ \begin{array}{c} (1-\alpha )f\prime ,for\;k=0\\ (1-4\sum_{m=1}^{M}w_{m}\sin^{2}\frac{\pi mk}{L})f^{\prime },for\;k=1,2,\ldots ,L-1 \end{array}\right.$$ (7)

in which *k* is the wave number of the Fourier mode (*λ*_*k*_ vs. *k* for different *α* values are shown in S4 Fig). The spatially uniform P1 state is stable when |*λ*_*k*_|<1 for any *k*. The stability of the 0-mode is the same as that of an uncoupled unit. Since *λ*_*k*_ for *k*>0 in Eq 7 does not depend on *α*, then the feedback has no effects on the stability of the uniform P1 state for non-zero mode. Because of this, the stability boundary separating uniform P1 from Dis-P2 appears to be a horizontal line independent of α (Fig 7A, solid).

*3) Stability of the Con-P2 state*. Following the same procedure as for the uniform P1 state, we obtained the eigenvalues for the spatially uniform P2 state as

$$\lambda_{k}=\left\{ \begin{array}{c} {\left(1-\alpha \right)}^{2}f_{1}^{\prime }f_{2}^{\prime },for\;k=0\\ {\left(1-4\sum_{m=1}^{M}w_{m}\sin^{2}\frac{\pi mk}{L}\right)}^{2}f_{1}^{\prime }f_{2}^{\prime },for\;k=1,2,\ldots ,L-1 \end{array}\right.$$ (8)

where $f_{1}^{\prime }$ and $f_{2}^{\prime }$ are the two derivatives of *f* at the P2 solution of Eq 5. Since the P2 solution depends on α, $f_{1}^{\prime }$ and $f_{2}^{\prime }$ are functions of α. Therefore, the stability boundary also depends on α (Fig 7A, dashed-dotted).

*4) Stability of the Dis-P2 states*. The stability of the Dis-P2 states cannot be analytically obtained. We used numerical simulations of the CML model (Eq 4) to determine the stability boundary (Fig 7A, dotted). No stable Dis-P2 patterns were obtained on the left side of the dotted line.

Spatiotemporal dynamics via numerical simulations of the CML model are also shown in Fig 7A, which are colored the same way as in Fig 3A and Fig 5A. The Dis-P2 only region exists between the uniform P1 stability boundary (solid line) and the Con-P2 stability boundary (dash-dotted line). The Con/Dis-P2 region exists between the Con-P2 stability boundary (dashed-dotted line) and the Dis-P2 stability boundary (dotted line). The Con-P2 only region exists between the uniform P1 stability boundary (dashed line) and the Dis-P2 stability boundary (dotted line). Note that the dotted line is almost identical to *α* = 0 except at the vicinity where all phases meet (inset in Fig 7A), indicating that stable Dis-P2 patterns can only exist when *α*>0. Histograms of domain size and example spatial patterns from three locations marked in Fig 7A are plotted (Fig 7B and 7C). The structure of the phase diagram and the statistical properties of the spatial patterns of the CML model match well with those of the generic FHN model and the ventricular myocyte model.

## Discussion

We investigated the roles of DGF in the genesis, selection, and stability of spatiotemporal patterns in periodically-paced excitable media. We used three models with different complexities, which allowed us to perform both numerical simulations and rigorous analytical treatments. The dynamical behaviors are well conserved in the three types of models, and the CML model reveals the dynamical mechanisms. Our major findings are as follows:

1. In the absence of DGF, both Con-P2 and Dis-P2 can occur depending on the pacing period and initial conditions. The Dis-P2 patterns are spatially random, determined by the initial conditions. The global signal (the spatial average) is a temporal P2 solution (alternans) with the alternans amplitude being randomly distributed between zero and the maximum amplitude (Con-P2).
2. In the presence of DGF, the pattern dynamics are determined by the sign of the DGF. When the DGF is negative, only Con-P2 patterns can exist, no spatial mode instabilities emerge, and all the Dis-P2 solutions are unstable. When the DGF is positive, both Con-P2 and Dis-P2 patterns can occur, depending on the pacing period and initial conditions. The Dis-P2 patterns are also spatially random but must satisfy that the global signals are temporal P1 solutions (no temporal alternans).
3. Bifurcation analyses of the CML model reveal the spatial-mode instabilities leading to the spatiotemporal patterns.
4. By linking the Ca^2+^-to-APD coupling properties to the DGF properties, we have shown that the spatiotemporal pattern dynamics of Ca^2+^ release in cardiac myocytes agree very well with the findings in the simple models, validating the theoretical predictions in a realistic system.

Therefore, our simulations and theoretical analyses reveal the underlying dynamical mechanisms and roles of DGF in the genesis, selection, and stability of spatiotemporal patterns in periodically-paced excitable media. The uniqueness of the conclusions drawn from the three types of models implies that the insights obtained in this study may apply to many excitable as well as oscillatory biological media. Here we discuss two examples below.

### A unified theory for subcellular Ca^2+^ alternans dynamics in cardiac myocytes

At the subcellular level, both Con-P2 and Dis-P2 Ca^2+^ release patterns (spatially concordant and discordant alternans) have been observed experimentally [31–33, 58, 59], but the underlying mechanisms remain incompletely understood. As shown in this study, the subcellular Ca^2+^ alternans dynamic of the ventricular myocyte model agree well with those of the simplified models, indicating that the generic mechanisms of pattern formation and selection are also applicable to Ca^2+^ alternans dynamics in cardiac myocytes. Both spatially concordant and discordant Ca^2+^ alternans (Con-P2 and Dis-P2 patterns) have been observed experimentally in cardiac myocyte [31–33, 59]. Shiferaw and Karma [60] developed a theory showing that a Turing instability caused by negative Ca^2+^-to-APD coupling is responsible for the formation of Dis-P2 patterns. A direct experimental demonstration of this theory was carried out by Gaeta et al. [33], who developed a method that could change the sign of Ca^2+^-to-APD coupling. However, Dis-P2 patterns have also been observed experimentally under voltage-clamp [32, 59] and free-running conditions without showing negative coupling [61]. Furthermore, previous simulation studies [44, 48] and this study have also shown that Con-P2 patterns can occur under negative Ca^2+^-to-APD coupling, and Dis-P2 patterns can occur under positive Ca^2+^-to-APD coupling and voltage-clamp conditions. These complex Ca^2+^ release behaviors cannot be well explained by the Turing instability mechanism alone. On the other hand, our study unifies the complex subcellular Ca^2+^ alternans dynamics under a single theoretical framework of DGF, providing a general mechanistic understanding of the subcellular Ca^2+^ alternans dynamics.

Previous studies [33, 48, 60] showed that the sign of Ca^2+^-to-APD coupling determines the occurrence of subcellular discordant Ca^2+^ alternans. One can achieve this by properly tuning the relative contributions of I_Ca_ and I_NCX_ [48, 49, 60]. Here we show that changing the maximum conductance I_SK_ or I_nsCa_ provides a more straightforward way of changing the coupling properties. Gaeta et al. [33] used a feedback control method to alter the coupling relationship and demonstrated in both simulations and experiments that the sign of the coupling controls the transitions between concordant and discordant Ca^2+^ alternans. However, our study shows that altering the Ca^2+^-to-APD coupling properties does not guarantee that one can control the transition between concordant and discordant Ca^2+^ alternans. The unique dynamical factor controlling the transition between concordant and discordant Ca^2+^ alternans is the sign of DGF, which is not uniquely determined by the sign of Ca^2+^-to-APD coupling. This is because Ca^2+^-to-APD coupling affects the recovery of I_Ca_ and SR Ca^2+^ loading which contribute oppositely to DGF.

### Applications to tissue-scale dynamics of excitable biological media

Cardiac and neural tissue are typical excitable media. In cardiac tissue, spatially concordant and discordant APD alternans have been widely observed in experiments [37, 62–64], and mechanisms have been revealed in mathematical modeling and theoretical studies [38, 65, 66]. However, in the real heart, electrical excitations cause mechanical contraction, and the contraction and stretch of the heart cells may result in the opening of the mechano-sensitive ion channels or alteration of intracellular Ca^2+^ cycling properties, a phenomenon called mechano-electric feedback [34–36]. Since contraction is a mechanical response of cardiac tissue, which is nearly a global signal, it is possible that the mechano-electric feedback can serve as global feedback or a DGF to affect the discordant APD alternans dynamics. This needs to be investigated in future studies using models with mechano-electric feedback [67–69].

In neural tissue, cluster firing and other firing patterns have been widely observed [70–73]. The role of delayed feedback or DGF in the formation of firing patterns has been investigated in computer models [39–41]. In a simulation study by Golomb and Rinzel [42], the authors showed similar Dis-P2 patterns of firing as the discordant patterns shown in our present study. We believe that the theoretical insights from our simple FHN model or the CML model will help shed light on the formation of the firing patterns.

### Links to pattern dynamics in oscillatory media with DGF

Our study focused on the roles of DGF in pattern formation and stability in periodically-paced excitable media. In a previous study in oscillatory chemical reaction experiments, Kim et al. [17] showed DGF caused clustering patterns similar to the Dis-P2 patterns in this study. Their observations were also demonstrated in computer simulations [19]. Since, in their studies, the DGF is an externally controlled signal, the delay time is a variable parameter. However, the DGF is intrinsic in the excitable biological media we investigated, and the delay time is simply the excitation period. Because of this, we can represent the system with a CML model that is able to capture the dynamics and the underlying bifurcations accurately. Since an excitable medium can become an oscillatory medium, the theories from our study may provide mechanistic insights into pattern dynamics of oscillatory media, such as the cluster patterning in the chemical reactions [14–19] or neural tissue [42].

## Methods

The present study involved three mathematical models at different levels of complexity. The model of a coupled array of FHN units and the 1D CML model are described in the Result section. A brief summary of the 3D ventricular cell model and numerical methods is given below.

### The 3D ventricular cell model

The ventricular cell model has been described in detail in our previous studies [44, 45], similar to other previous models [74–78]. Here we give a brief description of the model. The 3D cell model consists of 128×16×16 CRUs. Each CRU includes five sub-compartments: bulk cytosol, submembrane, dyad, junctional SR and network SR. The volumes of these sub-compartments are based on experimental data. The Ca^2+^ within a CRU cycles through these sub-compartments via diffusion, buffering/unbuffering, SR release and SERCA pump. The flow of Ca^2+^ between CRUs is via diffusion in the cytosol, submembrane and network SR. The exchange of Ca^2+^ between intracellular and extracellular space is regulated by I_Ca_ and Na^+^-Ca^2+^ exchanger (NCX). The model is described by differential equations that are coupled via Ca^2+^ and voltage. The closing and opening of L-type Ca^2+^ channels and the ryanodine receptors are simulated using stochastic Markov transitions. Therefore, intrinsic spatiotemporal noise exists in the model.

We added two new currents, *I*_*nsCa*_ and *I*_*SK*_, to the model for altering Ca^2+^-to-APD coupling. The *I*_*nsCa*_ formulation was adopted from the 1994 Luo and Rudy model [50] with the following parameter changes: P_ns(Ca)_ = 3.5×10^−5^ cm/s and K_m,ns(Ca)_ = 1.5 μM. *I*_*SK*_ was formulated based on Komendantov et al. [79] as follows:

$$I_{SK}=G_{SK}\frac{1}{1+{\left(\frac{K_{d}}{{\left[Ca^{2+}\right]}_{i}}\right)}^{4}}(V-E_{K}),$$ (9)

where *G*_*SK*_ = 0.005 mS/F and *K*_*d*_ = 0.6 μM.

The differential equation for voltage is then

$$C_{m}\frac{dV}{dt}=-(I_{Na}+I_{Ca,L}+I_{Ks}+I_{Kr}+I_{NCX}+I_{NaK}+I_{K1}+I_{to,f}+I_{to,s}+{I_{Cab}+\alpha_{nsCa}I_{nsCa}+\alpha_{SK}I}_{SK}+I_{sti})$$ (10)

where *C*_*m*_ = 1 *μF*/*cm*^2^ is the membrane capacitance. α_nsCa_ and α_SK_ are the parameters controlling the maximum conductance of *I*_*nsCa*_ and *I*_*SK*_, respectively. *I*_*sti*_ is the stimulus current density which is a square pulse with the amplitude -80 μA/μF and the duration 0.5 ms.

### Computer simulations and algorithms

Both the model of a coupled array of FHN units and the CML model were programmed with Python 3, and the corresponding simulations were carried out on our cluster with 24 Intel Xeon CPUs. The 3D ventricular cell model was programmed with CUDA C++, and the corresponding simulations were carried out on Nvidia Tesla K20c, K80, and GTX 1080 Ti GPU cards. The detailed algorithms for detecting spatiotemporal excitation patterns in this study are described in the S1 Text and S5 Fig.

## Supporting information

S1 Text The online supporting information includes: **A**. AP clamp and free running protocols. **B**. Stability analysis of the CML model. **C**. Automatic detection algorithms for spatiotemporal excitation patterns. **D**. Boundary between discordant P2 and uniform P2 in the CML model.
(DOCX)

S1 Fig Examples of spatiotemporal Ca^2+^ release dynamics in excitable biological systems. **a).** Data from Roome et al.[1] showing a spatiotemporal map of spontaneous dendritic voltage signal obtained from 5 s of linescan recording. Filled triangles indicate dendritic complex spikes (DCS). Subthreshold dendritic voltage modulation is also visible in the spatiotemporal voltage map. Red trace shows spatially averaged voltage, recorded at 2 kHz resolution. Corresponding spatiotemporal map for dendritic Ca^2+^ is also recorded. Green trace shows spatially averaged Ca^2+^. **b).** Data from Diaz et al.[2] showing spatiotemporal Ca^2+^ signals under control and ryanodine receptor blocker tetracaine with a voltage clamp protocol. Linescan recordings reveal out-of-phase spatial patterns of intracellular Ca^2+^ transient from beat to beat. Bottom two traces show fluorescence measured as a function of time at the regions indicated by arrows (i and ii).
(EPS)

S2 FigBifurcation diagrams for the FHN, detailed ventricular myocyte and CML models.**a).** Bifurcation diagram showing c vs. T for the single-element FHN model. **b).** Bifurcation diagram showing the peak values of the whole-cell average Ca^2+^ transient, ${\bar{c}}_{p}$ vs. T for the detailed ventricular myocyte model. **c).** Bifurcation diagram showing c vs. *γ* for the single-element CML model.(EPS)

S3 Fig Time trace of membrane voltage used in the AP clamp protocol for the detailed cell model. The voltage trace is the last action potential recorded in the simulation of the physiologically detailed myocyte model. The model was paced for 100 beats to reach the steady state at the pacing period T = 450 ms. This voltage trace was used in the AP clamp simulations.
(EPS)

S4 Fig *λ*_*k*_ vs. the wave number *k* for the 1D CML model under the P1 regime. *λ*_*k*_ vs. k for *α* = 0.2 (black), 0 (red) and -0.2 (blue) are numerically computed based on Eq 7 in the main text. *γ* = 1.057, which is the bifurcation point in S2C Fig.
(EPS)

S5 Fig Illustration of domain size detection by the pattern recognition algorithm. **a).** Top shows an example of discordant P2 patterns for the 1D FHN model. Bottom shows the relationship of *Δc*_*n*+1_(*j*) vs. j where the domain size *l* determined by the pattern recognition algorithm is marked by the double-arrow red line. **b)**. Same as a), but for the 1D CML model. **c).** Same as a), but for the detailed myocyte model. Note that the gray and black traces are for m = 1 and 50 in S10 Eq, respectively. We used m = 50 to extract the domain size for the detailed myocyte model in our study.
(EPS)
