# Supplementary material for: Delayed global feedback in the genesis and stability of spatiotemporal excitation patterns in paced biological excitable media
Source: PLoS Comput Biol. 2020 Oct 5;16(10):e1007931. doi: 10.1371/journal.pcbi.1007931 (PMC7561267; doi:10.1371/journal.pcbi.1007931)
Supplement: S1 Text — The online supporting information includes: A. AP clamp and free running protocols. B. Stability analysis of the CML model. C. Automatic detection algorithms for spatiotemporal excitation patterns. D. Boundary between discordant P2 and uniform P2 in the CML model. (DOCX) [file pcbi.1007931.s001.docx]

**Supplemental Information**

**Supplemental Text**

1. **AP clamp and free running protocols**

Two pacing protocols were used for the physiologically-detailed ventricular cell model: Free running and AP clamp. In the free running protocol, the cell is paced by a current pulse (I_sti_ in Eq.10 of main text) of -80 μA/μF and 0.5 ms. The voltage is described by the differential equation (Eq.10 in the main text). In the AP clamp protocol, the voltage is no longer described by the different equation but a pre-recorded input signal. In other words, in the computer program, instead of solving Eq.10 to obtain voltage, voltage is a time series input into the program. The voltage signal (S3 Fig) is the last action potential recorded in the simulation using the free running protocol, in which the model was paced for 100 beats to reach the steady state at the pacing period T=450 ms. This voltage waveform was repeated in time to give rise to a periodic signal and the period T was altered by altering (increasing or decreasing) the diastolic phase.

1. **Stability analysis of the CML model**

The equation of the 1D CML model is:

$c_{n+1}\left( j \right)=f\left[ c_{n}\left( j \right) \right]+\varepsilon\sum_{m=-M}^{M} w_{m}\{f\left[ c_{n}\left( j+m \right) \right]-f\left[ c_{n}\left( j \right) \right]\}-\alpha[f\left( \bar{c}_{n} \right)-f\left( \bar{c}_{s} \right)]$ (S1)

S1 Eq is the same as Eq.4 in the main text. Assuming $c_{n,s}(j)$ is the steady-state spatiotemporal solution, a small perturbation $\delta_{n,j}$ is applied to this state, i.e.,

$c_{n}\left( j \right)=c_{n,s}(j)+\delta_{n,j}$ (S2)

Linearization of S1 Eq around the steady-state gives rise to:

$\delta_{n+1,j}=f_{n,j}^{'}\delta_{n,j}+\varepsilon\sum_{m=-M}^{M} w_{m}(f_{n,j+m}^{'}-f_{n,j}^{'})\delta_{n,j}-\frac{\alpha}{L}\bar{f}_{n}^{'}\sum_{m=1}^{L} \delta_{n,m}$ (S3)

where $f_{n,j}^{'}=\left. \frac{df}{dc_{n}(j)} \right|_{c_{n}(j)=c_{n,s}(j)}$. For a spatially uniform steady-state solution, one can write S2 Eq as

$c_{n}\left( j \right)=c_{n,s}+\delta_{n}e^{i2\pi jk/L}$ (S4)

where *k*$\in${0, 1, …, L-1} is the wave number of the spatial Fourier modes. Inserting S4 Eq into S3 Eq, it becomes

$\delta_{n+1}e^{i2\pi jk/L}=f_{n}^{'}\delta_{n}e^{i2\pi jk/L}+2f_{n}^{'}\varepsilon\sum_{m=1}^{M} w_{m}\left( \cos\frac{2\pi mk}{L}-1 \right)\delta_{n}e^{i2\pi jk/L}-\frac{\alpha}{L}f_{n}^{'}\sum_{m=1}^{L} \delta_{n}e^{i2\pi mk/L}$ (S5)

S5 Eq can be reduced to

$\delta_{n+1}=f_{n}^{'}\delta_{n}+2f_{n}^{'}\varepsilon\sum_{m=1}^{M} w_{m}\left( \cos\frac{2\pi mk}{L}-1 \right)\delta_{n}-\frac{\alpha}{L}f_{n}^{'}\sum_{m=1}^{L} \delta_{n}e^{i2\pi(m-j)k/L}$ (S6)

The last term of S6 Eq can be further simplified, i.e.,

$$\frac{\alpha}{L}f_{n}^{'}\sum_{m=1}^{L} \delta_{n}e^{\frac{i2\pi\left( m-j \right)k}{L}}=\left\{ \begin{aligned} {\frac{\alpha}{L}f_{n}^{'}e}^{-\frac{i2\pi jk}{L}}\cdot\delta_{n}\cdot\sum_{m=1}^{L} e^{\frac{i2\pi mk}{L}}={\frac{\alpha}{L}f_{n}^{'}e}^{-\frac{i2\pi jk}{L}}\cdot\delta_{n}\cdot e^{\frac{i2\pi k}{L}}\cdot\frac{1-e^{\frac{i2\pi Lk}{L}}}{1-e^{\frac{i2\pi k}{L}}}=0, \\ for k\neq0 \\ \alpha f_{n}^{'}, for k=0 \end{aligned} \right.$$

Therefore, S6 Eq becomes

$\delta_{n+1}=\left\{ \begin{matrix} \left\{ f_{n}^{'}-4f_{n}^{'}\varepsilon\sum_{m=1}^{M} w_{m}\sin^{2} \frac{\pi mk}{L} \right\}\delta_{n}, for k>0 \\ f_{n}^{'}\left( 1-\alpha\right)\delta_{n}, for k=0 \end{matrix} \right.$ (S7)

From S7 Eq, one obtains the eigenvalues for uniform P1 as:

$\lambda_{k}=\left\{ \begin{matrix} (1-\alpha)f', for k=0 \\ \left( 1-4\sum_{m=1}^{M} w_{m}{sin}^{2} \frac{\pi mk}{L} \right)f^{'}, for k\in\{1, 2,\ldots, L-1\} \end{matrix} \right.$ (S8)

The eigenvalues for Con-P2 are:

$\lambda_{k}=\left\{ \begin{matrix} {(1-\alpha)}^{2}f_{1}^{'}f_{2}^{'}, for k=0 \\ {(1-4\sum_{m=1}^{M} w_{m}{sin}^{2} \frac{\pi mk}{L})}^{2}f_{1}^{'}f_{2}^{'}, for k\in\{1, 2, \ldots, L-1\} \end{matrix} \right.$ (S9)

1. **Automatic detection algorithms for spatiotemporal excitation patterns**

*1D array model of coupled FHN units*

To characterize the spatiotemporal excitation patterns, we defined the difference of the peak *c* of an FHN unit between two consecutive beats as

$\Delta c_{n+1}\left( j \right)=\left( -1 \right)^{n}\cdot\left[ c_{n+1}\left( j \right)-c_{n}\left( j \right) \right]$ (S10)

where n represents the beat number, j is the location index of the element in the 1D cable. Therefore, the node of an out-of-phase pattern is the location j where the sign of $\Delta c_{n+1}\left( j \right)$ switches. The domain size was measured between two neighboring nodes. Note that the term $\left( -1 \right)^{n}$ keeps the sign of $\Delta c_{n+1}\left( j \right)$ unchanged over n.

*CML model*

The same equation as S10 Eq is used to characterize the spatiotemporal excitation patterns in the CML model except that n represents the iteration number in the map model.

*Detailed ventricular myocyte model*

To overcome the noise in the spatiotemporal Ca^2+^ release patterns due to the intrinsic stochastic activity of ion channels in the model, we defined the average difference of peak Ca^2+^ transient of a CRU between two consecutive beats over the last *m* beats as

$\bar{\Delta c\left( j \right)}=\frac{1}{m}\sum_{n=N-m}^{N} \left( -1 \right)^{n}\cdot\left[ c_{n+1}\left( j \right)-c_{n}\left( j \right) \right]$ (S11)

where N=2000.

In summary, by using S10-S11 Eqs, we can identify spatially concordant (uniform) or discordant excitation patterns in all the three models. Given a discordant pattern, we can extract out-of-phase domain sizes of it. Examples of pattern recognitions using S10-S11 Eqs for FHN, CML, and detailed ventricular myocyte models are shown in S5 Fig.

1. **Boundary between discordant P2 and uniform P2 in the CML model**

The dotted line in Fig.7a is the simulated boundary between stable discordant P2 and unstable discordant P2. The protocol of the simulation is the following. We initially created a discordant P2 pattern with L/3 of the cable out-of-phase. We then added a noise term at each iteration as the following

$c_{n+1}\left( j \right)=f\left[ c_{n}\left( j \right) \right]+\varepsilon\sum_{m=-M}^{M} w_{m}\left\{ f\left[ c_{n}\left( j+m \right) \right]-f\left[ c_{n}\left( j \right) \right] \right\}+\alpha\left[ f\left( \bar{c}_{n} \right)-f\left( \bar{c}_{s} \right) \right]+\delta\xi_{n}\left( j \right)$ (S12)

where $\xi_{n}(j)$ is a random number drawn from a uniform distribution bounded within [-1,1] for the j^th^ element at n^th^ iteration, and $\delta$=0.0001 is the noise strength.

We simulated 2000 beats in the 1D cable for each set of coordinates in the ($\alpha, \gamma$) parameter space, where $\alpha$ and $\gamma$ were scanned from -0.02 to 0.05 and from 0.6 to 1.07, respectively. The patterns exhibited in the last two iterations for all these simulations were analyzed by the pattern recognition algorithm mentioned above. The boundary between discordant P2 and uniform P2 in the CML model was then determined by identifying the separation from discordant P2 to uniform P2 patterns.

**References**

1. Roome CJ, Kuhn B. Simultaneous dendritic voltage and calcium imaging and somatic recording from purkinje neurons in awake mice. Nat Commun. 2018;9: 3388. doi:10.1038/s41467-018-05900-3

2. Diaz ME, Eisner DA, O’Neill SC. Depressed ryanodine receptor activity increases variability and duration of the systolic Ca 2+ transient in rat ventricular myocytes. Circ Res. 2002;91: 585–593. doi:10.1161/01.res.0000035527.53514.c2
